# Magnetic resonance imaging‐based meniscal coverage and cartilage thickness are associated with mechanical axis alignment in knee osteoarthritis

**DOI:** 10.1002/jeo2.70927

**Published:** 2026-10-03

**Authors:** Atsushi Sasabe, Nobutake Ozeki, Hisako Katano, Yusuke Nakagawa, Tomomasa Nakamura, Hideyuki Koga, Ichiro Sekiya

**Affiliations:** ^1^ Center for Stem Cell and Regenerative Medicine, Institute of Science Tokyo Tokyo Japan; ^2^ Department of Cartilage Regeneration Institute of Science Tokyo Tokyo Japan; ^3^ Department of Joint Surgery and Sports Medicine Graduate School of Medical and Dental Sciences, Institute of Science Tokyo Tokyo Japan

**Keywords:** cartilage thickness, joint line convergence angle, knee osteoarthritis, magnetic resonance imaging, medial meniscus coverage

## Abstract

**Purpose:**

This study examined whether magnetic resonance imaging (MRI)‐based meniscal and cartilage measurements explain the percentage of mechanical axis (%MA) to a degree comparable with the joint line convergence angle (JLCA) when added to the arithmetic hip–knee–ankle (aHKA) angle. The association between tibial bony alignment and medial meniscal coverage across osteoarthritis (OA) severity was also assessed.

**Methods:**

This cross‐sectional study included 94 knees from 89 female patients (mean age, 54.6 years; Kellgren–Lawrence [KL] Grade 0–4). Weight‐bearing full‐leg radiographs were used to assess %MA, aHKA, the medial proximal tibial angle (MPTA) and JLCA. Three‐dimensional MRI measured medial meniscus coverage and cartilage thickness. Multiple regression compared the contributions of JLCA and MRI‐based measurements when each was added to aHKA. The MPTA‐by‐OA‐severity interaction was analyzed.

**Results:**

MPTA was significantly correlated with the medial meniscus coverage ratio (MMCR) in knees with advanced OA (KL Grade 3–4; *r* = 0.404) but not with mild or no OA (KL Grade 0–2; *r* = 0.064), whereas the MPTA‐by‐severity interaction was not significant (*p* = 0.116). Model A explained 67% of the %MA variance (adjusted *R* ^2^ = 0.668). Adding JLCA improved model fit (Model B: adjusted *R* ^2^ = 0.840). Adding MMCR and the height‐normalized medial‐to‐lateral cartilage thickness difference to aHKA achieved a comparable model fit (Model C: adjusted *R* ^2^ = 0.841), with all predictors significant (*p* < 0.01) and no multicollinearity. In a combined model including aHKA, JLCA, MMCR and cartilage thickness difference, all four variables remained significantly associated with %MA (adjusted *R* ^2^ = 0.864).

**Conclusion:**

Bony alignment and MRI‐based intra‐articular structural measurements were associated with weight‐bearing mechanical axis alignment in knee OA. When added to aHKA, MRI‐based meniscal coverage and cartilage thickness explained %MA with a model fit comparable to that obtained by adding JLCA. Thus, MRI‐based measurements may add complementary structural information to conventional radiographic evaluation.

**Level of Evidence:**

Level III, cross‐sectional study.

## Graphical abstract

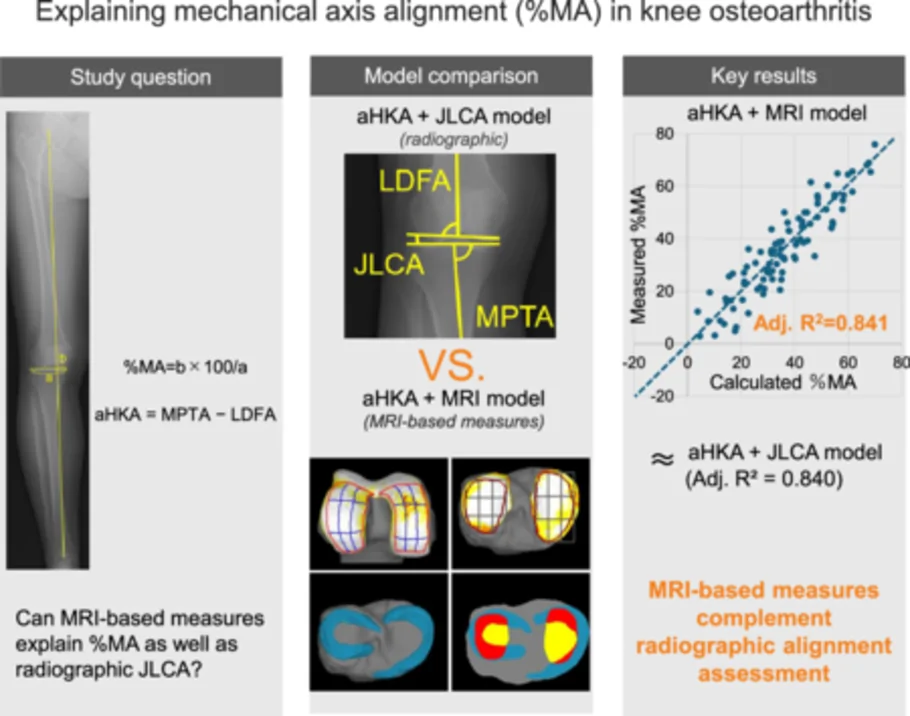

**Abbreviations**
aHKA: arithmetic hip–knee–ankle angle
BMI: body mass index
HTO: high tibial osteotomy
JLCA: joint line convergence angle
KL: Kellgren–Lawrence
LDFA: lateral distal femoral angle
LF: lateral femoral cartilage thickness
LT: lateral tibial cartilage thickness
MF: medial femoral cartilage thickness
MM: medial meniscus
MMCR: medial meniscus coverage ratio
MPTA: medial proximal tibial angle
MRI: magnetic resonance imaging
MT: medial tibial cartilage thickness
OA: osteoarthritis
PDW: proton density‐weighted
%MA: percentage of mechanical axis

## INTRODUCTION

Lower limb alignment is a key mechanical determinant of the initiation and progression of knee osteoarthritis (OA) [4, 7, 22]. It is widely determined using the percentage of mechanical axis (%MA); however, its measurement is influenced by intra‐articular structural changes. This has led to the proposal of another index of lower limb alignment, namely, the arithmetic hip–knee–ankle angle (aHKA) [12, 13].

The aHKA is the difference between the medial proximal tibial angle (MPTA) and the lateral distal femoral angle (LDFA) [12, 13]. When compared with the %MA, the aHKA is less affected by OA progression because it excludes the joint line convergence angle (JLCA) component, which reflects joint‐space asymmetry [12, 13]. However, discrepancies are observed between the aHKA and %MA values, and the factors underlying their relationship remain largely unexplained. One factor that may explain the discrepancies is the JLCA, because it may be influenced by ligamentous laxity, patient positioning and weight‐bearing conditions [11]. The asymmetry reflected by the JLCA can be assessed using magnetic resonance imaging (MRI) [15]; however, MRI is usually acquired under non‐weight‐bearing conditions, whereas the JLCA and %MA are assessed based on weight‐bearing radiographs. This suggests that altered joint loading needs to be considered when attempting to explain the discrepancies between %MA and the aHKA.

One consequence of altered joint loading is meniscal extrusion, which is an independent predictor of cartilage loss [3, 5, 23]. Detailed quantification of the types of changes in intra‐articular structures associated with OA is now possible using deep learning–based automatic segmentation and reconstruction of MRI data [1, 6, 9, 17, 18, 20, 21]. This approach can map cartilage thickness and volume for each knee subregion, thereby enabling an evaluation of the medial meniscus coverage ratio (MMCR), which is defined as the proportion of the medial tibial cartilage area covered by the meniscus [6]. However, because osteophytes at the tibial margin can hinder the automated measurement of meniscal extrusion, the MMCR is considered an area‐based measure of meniscal coverage that is conceptually related to, but not equivalent to, conventional linear extrusion measurements.

The associations between bony alignment indices (aHKA, LDFA and MPTA) and the MMCR have not been systematically examined in patients with knee OA. Furthermore, the relationships between %MA and either MMCR or cartilage thickness remain incompletely understood. Clarifying these associations could reveal whether bony alignment and intra‐articular soft‐tissue status are associated, rather than independent, contributors to limb alignment. The purpose of the present study was to examine whether MRI‐based meniscal and cartilage measurements can explain %MA to a degree comparable with JLCA when added to aHKA. A related aim was to assess the association between tibial bony alignment and medial meniscal coverage across OA severity. Two hypotheses were explored: (1) that the MRI‐based measurements can provide explanatory information comparable to JLCA, and (2) that the alignment‐coverage association is more evident in advanced OA.

## METHODS

### Participants

This cross‐sectional study was conducted at Tokyo Medical and Dental University Hospital (Institute of Science Tokyo). Consecutive female patients in their 50s who presented with knee pain between May 2020 and May 2023 and underwent three‐dimensional knee MRI were screened for eligibility. Women in their 50s constituted the largest single age‐ and sex‐specific group undergoing three‐dimensional knee MRI at this institution; therefore, restricting the study population to this group reduced heterogeneity related to age and sex while retaining a wide range of OA severity (KL Grades 0–4) and limb alignment. Knee MRI and weight‐bearing full‐leg anteroposterior radiography are routinely obtained as part of the evaluation of patients being considered for knee surgery, with MRI used to assess intra‐articular structures and full‐leg radiography used to assess lower limb alignment. The interval between the weight‐bearing radiographic and MRI examinations ranged from 1 to 4 weeks in all patients included in the final analysis. Accordingly, the cohort was not restricted to knees with definite radiographic OA but included symptomatic knees across KL Grades 0–4.

Each symptomatic knee meeting the eligibility criteria was used as the unit of analysis. Knees were excluded for the following reasons: absence of a standing weight‐bearing full‐leg radiograph or presence of a rotational knee deformity that precluded reliable radiographic alignment measurement (*n* = 22), history of previous knee surgery (*n* = 9), and severe varus malalignment (*n* = 2; defined as %MA < −20%), because these extreme values could exert disproportionate influence on the regression and correlation analyses (Figure 1). After applying these exclusion criteria, 94 knees from 89 patients were included in the final analysis. The potential effect of within‐patient correlation was also assessed by conducting a sensitivity analysis restricted to one knee per patient, with the first‐scanned knee selected for the five bilateral cases. All procedures were conducted in accordance with the Declaration of Helsinki, and the study was approved by the Medical Research Ethics Committee of the Institute of Science Tokyo (approval number: M2023‐088).

**Figure 1:**
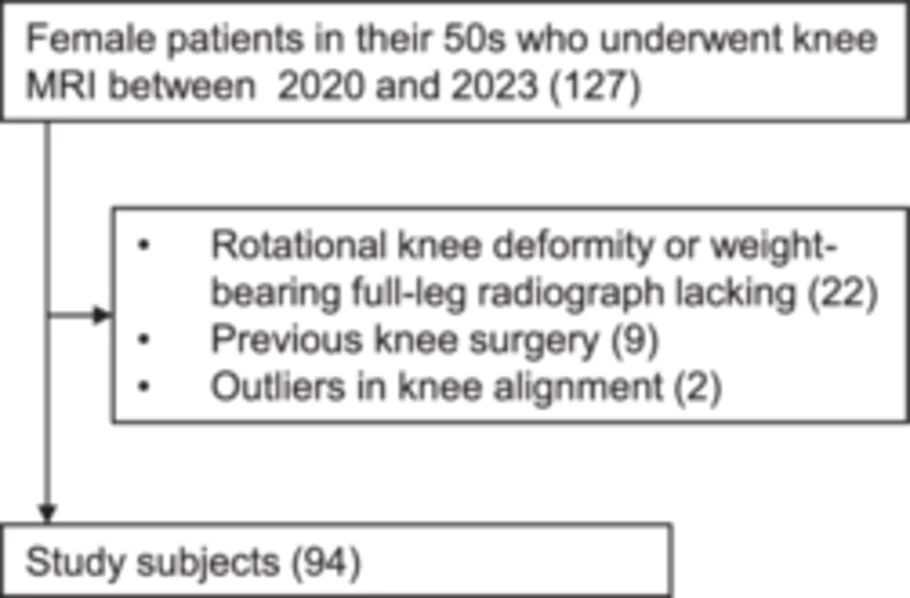
Flowchart of patient selection. Consecutive female patients in their 50s who underwent both weight‐bearing full‐leg radiography and knee MRI between May 2020 and May 2023 were identified. After the exclusions shown, 94 knees from 89 patients were included in the final analysis.

### Alignment measurement

Lower limb alignment was assessed using bilateral weight‐bearing full‐leg anteroposterior radiographs obtained with the patient standing in a standardized position with (i) the feet shoulder‐width apart and pointing forward, (ii) equal weight bearing on both limbs, and (iii) the patellae facing forward. All measurements were performed manually, using dedicated orthopaedic planning software, by a single trained observer who was blinded to the MRI‐based measurements and KL grade.

Five alignment indices were measured for the symptomatic knee of each patient using previously described standard methods [19]. The %MA was defined as the ratio of the distance from the medial edge of the tibial plateau to the point where the mechanical axis intersects the tibial plateau, divided by the total width of the tibial plateau and expressed as a percentage. The LDFA was defined as the angle between the femoral mechanical axis and the distal femoral joint line. The MPTA was defined as the angle between the tibial mechanical axis and the proximal tibial joint line. The JLCA was defined as the angle between the distal femoral joint line and the proximal tibial joint line and reflected intra‐articular joint space asymmetry. The aHKA was calculated as MPTA minus LDFA and represents the articular alignment independently of overall limb geometry [12, 13] (Figure 2).

**Figure 2:**
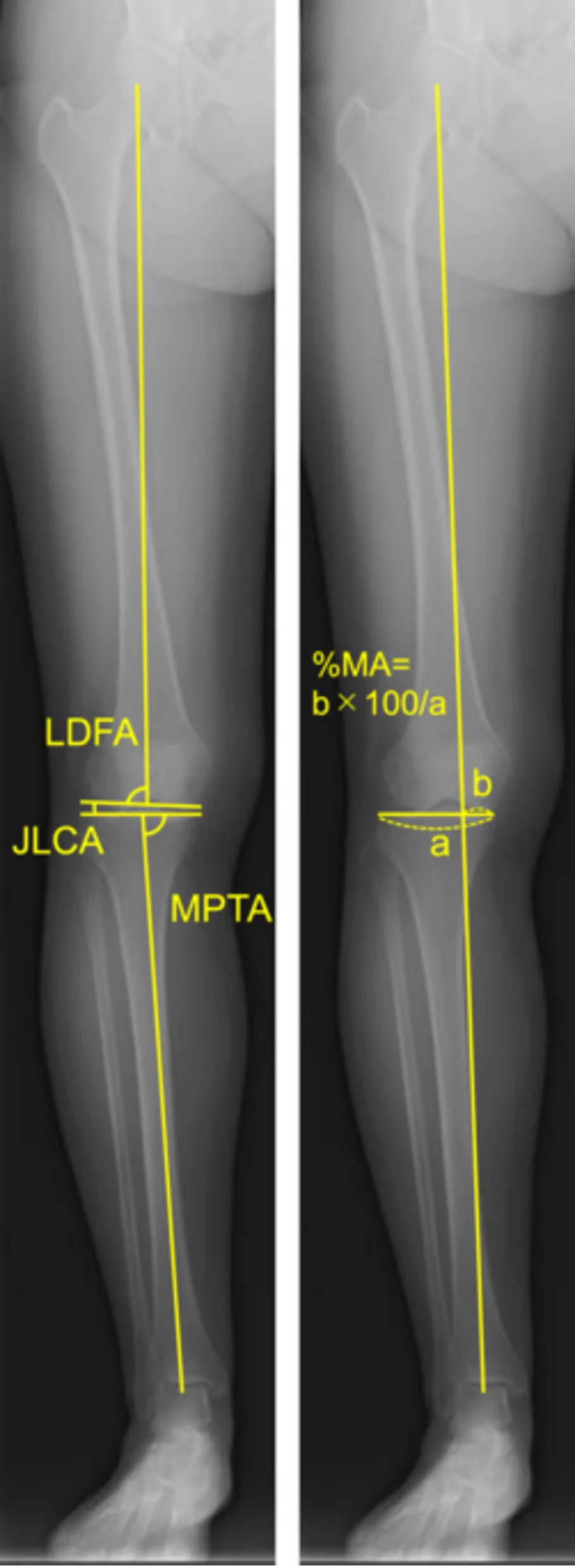
Measurement of radiographic alignment indices on weight‐bearing full‐leg anteroposterior radiographs. MPTA (medial proximal tibial angle): the medial angle between the tibial mechanical axis and the proximal tibial joint line. LDFA (lateral distal femoral angle): the lateral angle between the femoral mechanical axis and the distal femoral joint line. JLCA (joint line convergence angle): the angle between the distal femoral joint line and the proximal tibial joint line, representing intra‐articular joint space asymmetry. %MA (percentage of mechanical axis): the point where the mechanical axis (a line from the centre of the femoral head to the centre of the ankle talus) intersects the proximal tibia, expressed as a percentage of the tibial plateau width from the medial edge (0% = medial edge, 100% = lateral edge); a value of 50% indicates neutral alignment, with lower values indicating varus and higher values indicating valgus alignment. The arithmetic hip–knee‐ankle angle (aHKA) was calculated as MPTA − LDFA and is not shown.

The intra‐observer reliability of the radiographic measurements was assessed in 20 randomly selected knees, which were remeasured by the same observer after an interval of at least two weeks. The observer was blinded to the initial measurements. Intra‐observer reliability was evaluated using the intraclass correlation coefficient (ICC; two‐way mixed‐effects model, single measures). The inter‐observer reliability of these radiographic measurements at this institution has been reported previously and showed excellent agreement (ICCs, 0.91–0.99) [18]. As the aHKA is calculated from the MPTA and LDFA, its inter‐observer reliability is supported by the high reliability of these two measurements.

### MRI protocol and measurements

MRI was performed at 3.0 T (Achieva 3.0TX, Philips) using a dedicated 16‐channel transmit/receive knee coil (dStream T/R Knee 16ch coil, Philips). A three‐dimensional sagittal proton density‐weighted (PDW) sequence was acquired with an acquisition voxel size of 0.6 × 0.6 × 0.6 mm, reconstructed to 0.3 × 0.3 × 0.3 mm. The slice thickness was 0.3 mm with no interslice gap, and a total of 320 slices were obtained per knee. The field of view was 150 × 150 mm, and the total scan time was 7 min 10 s.

MRI analyses were performed using a three‐dimensional image analysis system (SYNAPSE 3D [Japanese version: SYNAPSE VINCENT], collaborative version, Fujifilm). The validity and reproducibility of this automated 3D MRI analysis system have been previously reported [1, 6, 9, 17, 18, 20, 21]. A previous validation of this system reported high segmentation accuracy (dice similarity coefficients: 0.89–0.92 for cartilage and the medial meniscus [MM]) [1]. In the present cohort, the automated segmentation results were visually inspected for all knees, with no manual corrections performed. The meniscal and bone regions were automatically segmented from PDW images and reconstructed into three‐dimensional models. The femoral and tibial cartilage regions were displayed in vertical projections and automatically divided into medial and lateral regions based on the ROI shape. The ROIs were automatically defined from the predicted bone morphology using the trained neural network to ensure that clear osteophytes were excluded from the ROIs, whereas cartilage defects and severe cartilage thinning within the ROIs were reflected in the area‐based measurements.

Meniscal segmentation was performed based on the meniscal morphology identified by the automated algorithm; meniscal tears, including root tears, were not separately classified or incorporated as independent variables in the present analysis. Cartilage thickness was automatically calculated as the mean thickness within each medial and lateral ROI for both the femur and tibia (Figure 3A,B).

**Figure 3:**
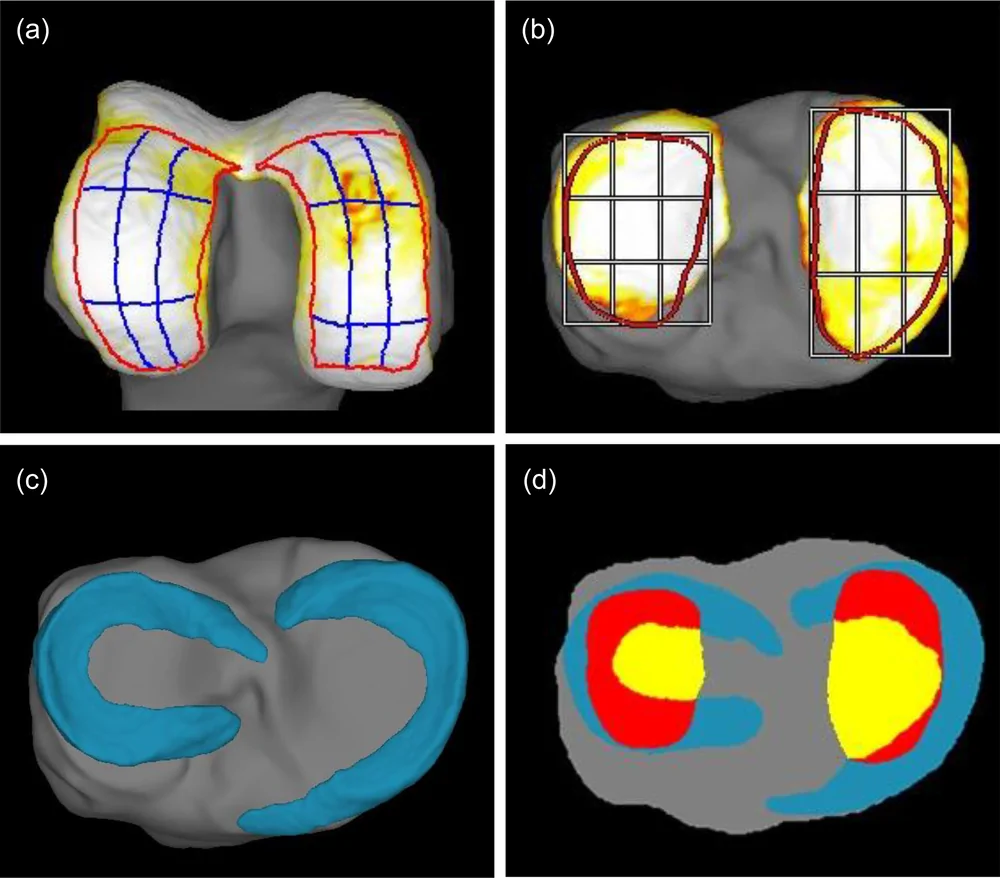
Automated three‐dimensional (3D) MRI measurement of cartilage thickness and medial meniscus coverage. Representative 3D MRI images demonstrating the automated measurement procedures used in this study. (A) Automated segmentation of femoral cartilage on 3D MRI. The region of interest (ROI) was automatically defined by the software (red outline), and femoral cartilage thickness was calculated automatically within the ROI. (B) Automated segmentation of tibial cartilage on 3D MRI. The tibial cartilage ROI was automatically delineated (red outline), and tibial cartilage thickness was measured automatically. (C) Automated three‐dimensional extraction of the meniscus from 3D MRI. The meniscal volume was segmented automatically without manual intervention. (D) Schematic illustration of the medial meniscus coverage ratio. The tibial cartilage ROI is indicated by the combined red and yellow area. The red area represents the portion of the tibial cartilage covered by the meniscus, and the yellow area represents the uncovered portion. The medial meniscus coverage ratio was calculated as the ratio of the meniscus‐covered tibial cartilage area (red) to the total tibial cartilage ROI area (red + yellow). MRI, magnetic resonance imaging.

The long axis of the tibia was established as a reference axis. The MM, including its anterior and posterior horns, was projected onto a plane perpendicular to the tibial long axis. The medial tibial cartilage ROI was similarly projected onto the same plane. The MMCR was calculated as the ratio of the total medial tibial cartilage ROI area covered by the projected MM area for each knee (Figure 3C,D). Because MMCR is an area‐based measure of meniscal coverage of the tibial cartilage rather than a direct measure of meniscal extrusion, a lower MMCR may reflect meniscal displacement, reduced or altered meniscal size, differences in tibial cartilage area or a combination of these factors.

### Statistical analysis

All statistical analyses were performed using Bell Curve for Excel (Social Survey Research Information Co., Ltd.). Differences in demographic, radiographic, and MRI‐based variables across KL grades were assessed using one‐way analysis of variance for the five KL grades (0–4) and an unpaired Welch *t* test comparing the KL Grades 0–2 and 3–4 groups (Table S1). Pearson's product‐moment correlation coefficients were calculated to examine the associations between alignment indices (%MA, LDFA, MPTA and aHKA) and MRI‐based measurements (MMCR, medial and lateral femoral cartilage thickness, and medial and lateral tibial cartilage thickness) for the overall cohort (KL Grades 0–4, *n* = 94) and for two KL grade subgroups (KL Grades 0–2, *n* = 61; KL Grades 3–4, *n* = 33). The associations of MPTA and LDFA with MMCR in the overall cohort were designated as the two primary correlation analyses. To account for multiple testing of these two primary analyses, a Bonferroni‐corrected significance threshold of *p* < 0.025 was applied, and 95% confidence intervals were calculated using Fisher's *z*‐transformation. Correlation analyses within the KL grade subgroups and the remaining correlations were considered exploratory. The difference in the MPTA–MMCR association between the KL Grades 0–2 and 3–4 subgroups was examined using an MPTA‐by‐severity interaction term.

Linear regression analyses were performed with measured %MA as the dependent variable to examine the independent contributions of bony alignment and MRI‐based soft‐tissue variables. Three models were constructed: Model A (aHKA alone; simple linear regression), Model B (aHKA+JLCA), and Model C (aHKA + height‐normalized cartilage difference + MMCR). Models B and C shared aHKA as a common base and differed only in terms of whether JLCA or the MRI‐based variables were added. In addition, a combined model including aHKA, JLCA, the height‐normalized cartilage difference and MMCR was constructed to examine whether JLCA and the MRI‐based variables provided independent contributions to %MA. The height‐normalized cartilage difference was defined as follows: ([medial femoral cartilage thickness + medial tibial cartilage thickness] − [lateral femoral cartilage thickness + lateral tibial cartilage thickness])/height. Height was expressed in metres, and cartilage thicknesses in millimetres; therefore, the unit was mm/m. The medial‐minus‐lateral difference represented the imbalance in cartilage thickness between compartments. Division by height was used to reduce the potential influence of body size on absolute cartilage thickness measurements.

Model fit was evaluated using the adjusted coefficient of determination (adjusted *R* ^2^), and multicollinearity was assessed using the variance inflation factor (VIF), with values below 10 considered acceptable. Sensitivity analyses were performed by restricting the dataset to one knee per patient (*n* = 89), excluding the six knees with KL Grade 4, and using Spearman's rank correlation to assess the robustness of the Pearson correlation results to potential deviations from normality (Table S2). For the five bilateral cases, the first‐scanned knee was selected; all five had KL Grades 0–2. For all other analyses, statistical significance was set at *p* < 0.05, with exploratory analyses interpreted cautiously in view of the multiple testing performed.

## RESULTS

The final cohort consisted of 94 knees from 89 patients, including five patients with bilateral knees. All five bilateral cases had KL Grades 0–2. The cohort comprised 26 KL Grade 0, 20 KL Grade 1, 15 KL Grade 2, 27 KL Grade 3 and 6 KL Grade 4 knees. The mean age, height and BMI of the study population were 54.6 years, 158.1 cm and 25.0 kg/m^2^, respectively, and these characteristics were generally comparable across the KL grade subgroups. The mean %MA of all included knees was 36.0% and was significantly lower in the KL Grades 3–4 group (30.3%) than in the KL Grade 0–2 group (39.2%), indicating increasing varus malalignment with advancing OA severity. The mean MPTA did not differ significantly across KL grades (range, 85.5–86.4°), whereas LDFA remained relatively stable (range, 87.3–88.0°). The JLCA increased significantly with advancing OA severity, from 0.3° in KL Grade 0 to 3.1° in KL Grade 4 (Table 1). The intra‐observer reliability of the radiographic measurements was excellent for all parameters: %MA (ICC = 0.997; 95% CI, 0.994–0.999), LDFA (ICC = 0.962; 95% CI, 0.906–0.985), MPTA (ICC = 0.923; 95% CI, 0.817–0.969), JLCA (ICC = 0.931; 95% CI, 0.834–0.972) and aHKA (ICC = 0.938; 95% CI, 0.850–0.975).

**Table 1:** Patient demographics, radiographic alignment indices and MRI‐based meniscal and cartilage measurements stratified by Kellgren–Lawrence (KL) grade.

Demographics and radiographic alignment indices								
	*n*	Height	Age	BMI	%MA	LDFA	MPTA	JLCA
KL0	26	157.2 ± 5.8	54.4 ± 2.7	24.6 ± 5.5	40.1 ± 17.3	87.7 ± 3.2	86.4 ± 2.2	0.3 ± 1.1
KL1	20	157.9 ± 5.2	54.3 ± 3.0	25.3 ± 4.7	39.2 ± 14.0	87.5 ± 1.9	86.3 ± 2.0	0.6 ± 1.5
KL2	15	160.8 ± 6.1	54.0 ± 2.0	23.9 ± 3.3	37.5 ± 10.9	87.3 ± 1.5	86.1 ± 2.1	1.0 ± 2.1
KL3	27	157.6 ± 6.6	55.5 ± 3.0	26.0 ± 4.5	31.3 ± 19.2	88.0 ± 2.0	85.6 ± 1.7	2.0 ± 2.5
KL4	6	159.1 ± 4.8	54.4 ± 3.1	25.1 ± 3.1	25.9 ± 13.6	87.7 ± 0.5	85.5 ± 1.7	3.1 ± 2.1
KL0–4	94	158.1 ± 6.0	54.6 ± 2.9	25.0 ± 4.6	36.0 ± 16.8	87.7 ± 2.3	86.0 ± 2.0	1.2 ± 2.1

MRI‐based meniscal and cartilage measurements								
	*n*	MMCR	F	MF	LF	T	MT	**LT**
KL0	26	0.48 ± 0.10	1.9 ± 0.2	1.9 ± 0.2	1.8 ± 0.3	2.1 ± 0.3	1.9 ± 0.3	2.4 ± 0.3
KL1	20	0.41 ± 0.13	2.0 ± 0.2	1.9 ± 0.3	1.9 ± 0.2	2.2 ± 0.3	2.0 ± 0.3	2.4 ± 0.4
KL2	15	0.41 ± 0.12	2.1 ± 0.2	2.0 ± 0.3	2.0 ± 0.2	2.2 ± 0.2	1.9 ± 0.4	2.5 ± 0.4
KL3	27	0.41 ± 0.15	2.0 ± 0.2	1.7 ± 0.4	2.0 ± 0.4	2.1 ± 0.4	1.7 ± 0.5	2.5 ± 0.6
KL4	6	0.26 ± 0.18	1.9 ± 0.1	1.6 ± 0.5	2.1 ± 0.3	1.9 ± 0.1	1.4 ± 0.5	2.4 ± 0.4
KL0–4	94	0.42 ± 0.14	2.0 ± 0.2	1.9 ± 0.3	1.9 ± 0.3	2.1 ± 0.3	1.8 ± 0.4	2.4 ± 0.5

*Note*: Data are presented as mean ± standard deviation (SD). Group comparisons between KL grades are provided in Table S1.

Abbreviations: BMI, body mass index; F, femoral cartilage thickness; JLCA, joint line convergence angle; LDFA, lateral distal femoral angle; LF, lateral femoral cartilage thickness; LT, lateral tibial cartilage thickness; MF, medial femoral cartilage thickness; MMCR, medial meniscus coverage ratio; MPTA, medial proximal tibial angle; MRI, magnetic resonance imaging; MT, medial tibial cartilage thickness; T, tibial cartilage thickness; %MA, percentage of mechanical axis.

The MMCR decreased significantly across the five KL grades, from 0.48 in KL Grade 0 to 0.26 in KL Grade 4, indicating an increasing loss of medial tibial plateau coverage with increasing OA severity. The medial femoral (MF) and medial tibial (MT) cartilage thicknesses were significantly thinner in the KL Grades 3–4 group (MF, 1.71 mm; MT, 1.65 mm) than in the KL Grades 0–2 group (MF, 1.95 mm; MT, 1.92 mm) (Table S1).

The prespecified primary correlations were MPTA–MMCR and LDFA–MMCR (Table 2), with a Bonferroni‐corrected significance threshold of *p* < 0.025. All remaining correlations were considered secondary or exploratory and were interpreted without formal adjustment for multiple comparisons. In the overall cohort (KL Grades 0–4, *n* = 94), LDFA was not correlated with MMCR (*r* = 0.037; 95% CI, −0.17 to 0.24), whereas MPTA showed a positive correlation with MMCR (*r* = 0.216; 95% CI, 0.01–0.40) that did not meet the Bonferroni‐corrected significance threshold. Among the secondary analyses, %MA showed positive correlations with MMCR (*r* = 0.301), MF cartilage thickness (*r* = 0.435) and MT cartilage thickness (*r* = 0.491), while MPTA was correlated with MF (*r* = 0.232) and MT (*r* = 0.273).

**Table 2:** Pearson correlation coefficients between radiographic alignment indices and MRI‐based meniscal and cartilage measurements for (A) all knees (KL Grades 0–4, *n* = 94), (B) KL Grades 0–2 (*n* = 61) and (C) KL Grades 3–4 (*n* = 33).

KL Grades 0–4, *n* = 94					
	MMCR	MF	LF	MT	LT
%MA	0.301 (0.003)	0.435 (<0.001)	−0.064 (0.541)	0.491 (<0.001)	−0.187 (0.072)
LDFA	0.037 (0.722)	0.035 (0.741)	0.024 (0.819)	0.013 (0.904)	0.060 (0.566)
MPTA	0.216 (0.037)*	0.232 (0.025)	0.041 (0.696)	0.273 (0.008)	0.068 (0.514)
aHKA	0.119 (0.252)	0.132 (0.204)	0.010 (0.925)	0.177 (0.088)	0.001 (0.992)
Age	−0.117 (0.260)	−0.173 (0.095)	−0.037 (0.721)	−0.171 (0.099)	0.075 (0.470)

KL Grades 0–2, *n* = 61					
	MMCR	MF	LF	MT	LT
%MA	0.110 (0.397)	0.162 (0.214)	0.303 (0.018)	0.318 (0.013)	0.162 (0.213)
LDFA	0.124 (0.342)	0.079 (0.547)	−0.109 (0.404)	0.011 (0.933)	−0.069 (0.595)
MPTA	0.064 (0.623)	0.100 (0.444)	0.324 (0.011)	0.247 (0.055)	0.309 (0.015)
aHKA	−0.054 (0.680)	0.007 (0.959)	0.312 (0.014)	0.163 (0.210)	0.270 (0.035)
Age	−0.201 (0.121)	−0.075 (0.568)	−0.162 (0.213)	−0.179 (0.168)	0.044 (0.735)

KL Grades 3–4, *n* = 33					
	MMCR	MF	LF	MT	LT
%MA	0.431 (0.012)	0.597 (<0.001)	−0.330 (0.060)	0.572 (<0.001)	−0.531 (0.001)
LDFA	−0.064 (0.724)	0.052 (0.772)	0.206 (0.251)	0.085 (0.638)	0.270 (0.129)
MPTA	0.404 (0.020)	0.331 (0.060)	−0.288 (0.104)	0.249 (0.163)	−0.267 (0.134)
aHKA	0.312 (0.077)	0.181 (0.312)	−0.335 (0.056)	0.104 (0.565)	−0.367 (0.036)
Age	0.045 (0.802)	−0.183 (0.309)	0.015 (0.935)	−0.084 (0.641)	0.110 (0.541)

*Note*: Values are presented as Pearson correlation coefficients (*r*) with *p* values in parentheses. The prespecified primary correlations were MPTA–MMCR and LDFA–MMCR, for which a Bonferroni‐corrected significance threshold of *p* < 0.025 was applied. All other correlations were considered secondary or exploratory and were interpreted without formal adjustment for multiple comparisons.

Abbreviations: aHKA, arithmetic hip–knee–ankle angle; KL, Kellgren–Lawrence; LDFA, lateral distal femoral angle; LF, lateral femoral cartilage; LT, lateral tibial cartilage; MMCR, medial meniscus coverage ratio; MF, medial femoral cartilage; MPTA, medial proximal tibial angle; MT, medial tibial cartilage; %MA, percentage of mechanical axis.

* Significant at *p* < 0.05 but not after Bonferroni correction (primary analysis; threshold *p* < 0.025).

MPTA showed little evidence of an association with MMCR in the KL Grades 0–2 subgroup (*n* = 61; *r* = 0.064; 95% CI, −0.19 to 0.31) (Figure 4a). In the KL Grades 3–4 subgroup (*n* = 33), MPTA showed a positive correlation with MMCR (*r* = 0.404; 95% CI, 0.07–0.66) (Figure 4b). Sensitivity analyses that excluded the six KL Grade 4 knees and used Spearman rank correlation yielded results generally consistent with the primary analysis (Table S2). However, a regression model that included an MPTA‐by‐severity interaction term showed no statistically significant interaction (*p* = 0.116). Therefore, the apparent difference in the MPTA–MMCR association between the two severity strata should be regarded as exploratory. The aHKA showed no clear correlations with any MRI outcome in the overall group.

**Figure 4:**
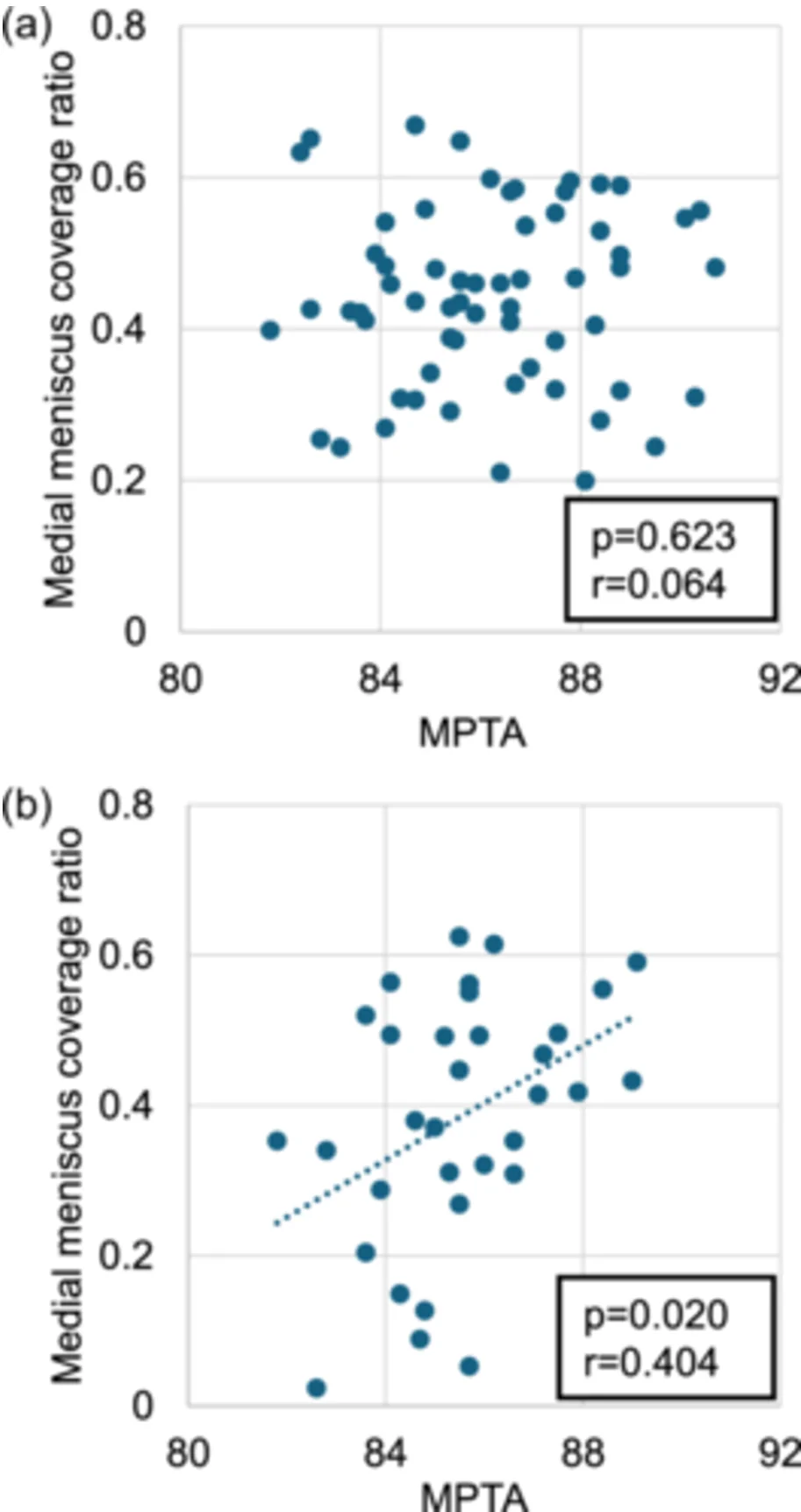
Association between the medial proximal tibial angle (MPTA) and the medial meniscus coverage ratio stratified by OA severity. Scatter plots showing the association between the MPTA and the medial meniscus coverage ratio in knees with Kellgren–Lawrence (KL) Grades 0–2 (a) and KL Grades 3–4 (b). Each data point represents one knee. In (b) (KL Grades 3–4), a dashed regression line is shown, reflecting a statistically significant correlation between MPTA and medial meniscus coverage ratio. No regression line is shown in (a) (KL Grades 0–2), as no significant correlation was observed. The Pearson's product–moment correlation coefficient (*r*) and *p* value are displayed in each panel. OA, osteoarthritis.

Linear regression analyses were performed using measured %MA as the dependent variable. Model A, using aHKA alone, yielded the equation %MA = 4.63 × aHKA + 43.8 (adjusted *R* ^2^ = 0.668), demonstrating that aHKA alone explained approximately 67% of the variance in the measured %MA (Figure 5a). Model B, which also incorporated JLCA, significantly improved the model fit, yielding the equation %MA = 4.25 × aHKA − 3.37 × JLCA + 47.0 (adjusted *R* ^2^ = 0.840) (Figure 5b). Model C, which incorporated MRI‐based measurements (height‐normalized cartilage difference and MMCR) in addition to aHKA, yielded the equation %MA = 4.32 × aHKA + 19.6 × height‐normalized cartilage difference + 13.8 × MMCR + 42.0 (adjusted *R* ^2^ = 0.841) (Figure 5c). All predictors in Models B and C were statistically significant (*p* < 0.01), and the VIF values were below 2.0 in Models A–C, indicating no substantial multicollinearity.

**Figure 5:**
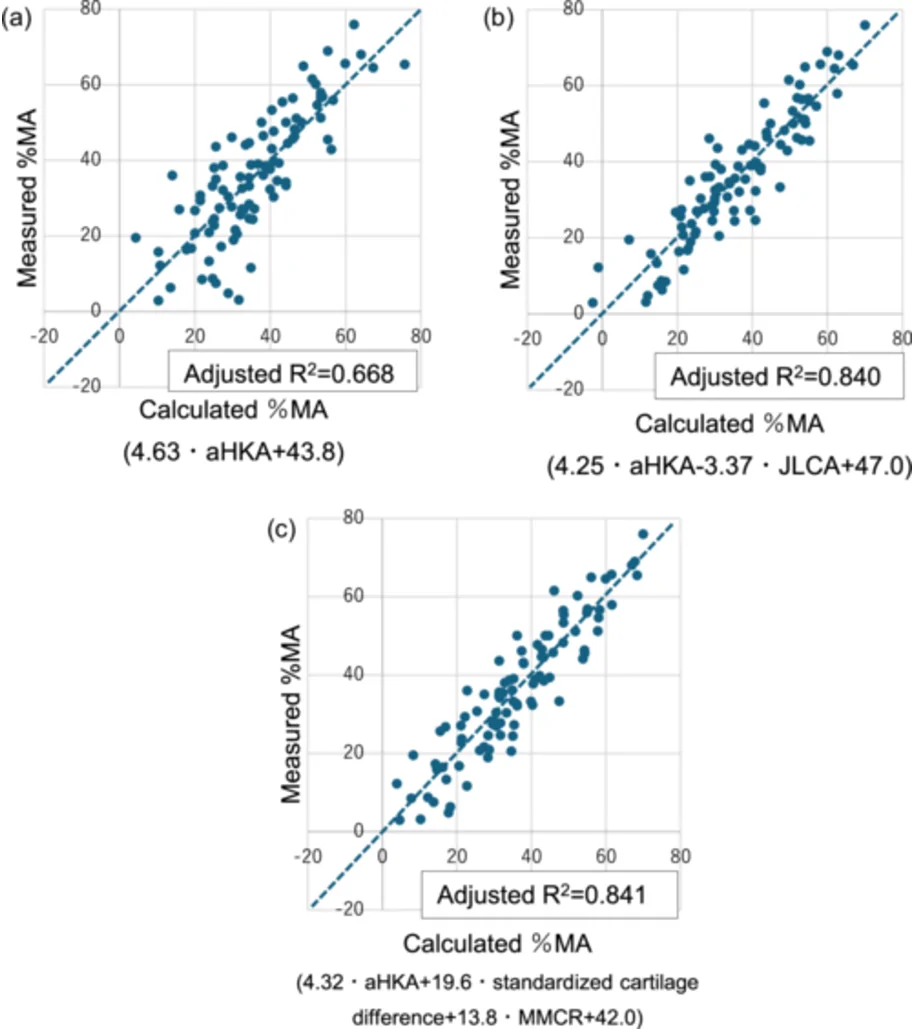
Agreement between calculated and measured percentage of mechanical axis (%MA) for the three regression models. Scatter plots showing the agreement between calculated and measured %MA. Measured %MA (vertical axis) was obtained directly on weight‐bearing full‐leg anteroposterior radiographs, and calculated %MA (horizontal axis) was derived from each regression equation. Each data point represents one knee, and the dashed line indicates perfect agreement (*y* = *x*). The adjusted R‐squared (adjusted *R* ^2^) is shown in each panel. (a) Calculated %MA derived from aHKA alone: %MA = 4.63 × aHKA + 43.8. (b) Calculated %MA derived from aHKA and JLCA: %MA = 4.25 × aHKA − 3.37 × JLCA + 47.0. (c) Calculated %MA derived from aHKA combined with MRI‐based cartilage and meniscal measurements: %MA = 4.32 × aHKA + 19.6 × height‐normalized cartilage difference + 13.8 × MMCR + 42.0, where height‐normalized cartilage difference was defined as ([medial femoral cartilage thickness + medial tibial cartilage thickness] − [lateral femoral cartilage thickness + lateral tibial cartilage thickness])/height, and MMCR denotes medial meniscus coverage ratio. MRI, magnetic resonance imaging.

A combined model that included aHKA, JLCA, MMCR and the height‐normalized cartilage difference was also constructed. All four predictors remained statistically significant (each *p* < 0.05), and the model achieved an adjusted *R* ^2^ of 0.864. The VIF values remained below 2.5, with values of approximately 2.3 for JLCA and the height‐normalized cartilage difference.

The primary findings remained stable when the analysis was restricted to one knee per patient (*n* = 89), indicating that inclusion of bilateral knees did not materially affect the results. Spearman rank correlation analyses also produced similar results (Table S2). Exclusion of the six knees with KL Grade 4 likewise yielded results generally consistent with the primary analyses (Table S2).

The findings also remained stable when the two knees with severe varus malalignment (%MA < −20%) were included. The correlation between MPTA and MMCR was *r* = 0.379 versus *r* = 0.216 in the primary analysis, and the adjusted *R* ^2^ of Model C was 0.875 versus 0.841 in the primary analysis. Together, these sensitivity analyses indicate that the main findings were not materially influenced by bilateral observations, correlation method, the small number of KL Grade 4 knees or exclusion of the two extreme varus cases.

## DISCUSSION

This cross‐sectional study investigated the relationships between lower limb alignment indices and MRI‐based structural measurements in 94 knees across the full spectrum of OA severity. Two principal findings emerged: MPTA was positively correlated with MMCR in the KL Grades 3–4 subgroup, whereas little evidence of an association was observed in the KL Grades 0–2 subgroup. However, the interaction with OA severity was not statistically significant; therefore, this subgroup finding should be considered exploratory.

The MRI‐based meniscal and cartilage measurements provided explanatory information for the measured %MA beyond aHKA alone. When added to aHKA, these measurements achieved a model fit comparable to that obtained by adding JLCA (adjusted *R* ^2^, 0.841 vs. 0.840). These findings formed the second principal observation of the present study.

Model A, which included aHKA alone, achieved an adjusted *R* ^2^ of 0.668. This relatively high model fit should not be interpreted as evidence of a novel biological association. Because aHKA reflects bony coronal alignment and %MA represents the position of the weight‐bearing mechanical axis, part of their association likely reflects their inherent geometric and anatomical relationships.

Model B, which combined aHKA and JLCA, increased the adjusted *R* ^2^ to 0.840. This high model fit is partly expected because %MA, aHKA, and JLCA are geometrically related measurements derived from the same weight‐bearing radiograph. Therefore, their strong statistical relationships may partly reflect their shared geometric derivation rather than independent biological information. Accordingly, the high adjusted *R* ^2^ of Model B should not be interpreted as evidence of an independent biological relationship among these radiographic parameters.

Model C, which combined aHKA with MMCR and the height‐normalized cartilage difference, achieved a similar model fit (adjusted *R* ^2^ = 0.841). The more relevant finding is that adding MRI‐based meniscal and cartilage measurements to the aHKA provided explanatory information beyond the aHKA alone, with a model fit similar to that obtained by adding JLCA. Furthermore, when aHKA, JLCA, MMCR and the height‐normalized cartilage difference were entered together in the combined model, all four predictors remained statistically significant. These findings suggest that the MRI‐based measurements provide information that partly overlaps with, but is not fully captured by, conventional radiographic alignment measures and may therefore offer complementary structural information regarding measured %MA. This interpretation should be considered in the context of previous reports linking lower limb alignment with medial meniscal extrusion.

Lower limb alignment has been associated with medial meniscal extrusion in previous studies. For example, Goto et al. [5] demonstrated that alignment indices, including MPTA, were associated with medial meniscal extrusion in knees with OA, while Shen et al. [23] similarly reported that lower limb alignment parameters provided independent predictive value for the severity of medial meniscal extrusion. Tsushima et al. [24] further reported that, among radiographic alignment parameters, the JLCA was the parameter most strongly associated with both the severity of medial knee OA and medial meniscal extrusion. Consistent with these clinical observations, a finite element analysis demonstrated that medial meniscal extrusion and the varus tilt of the JLCA increase the stress arising in the medial compartment [10]. The present findings are broadly consistent with these observations, although the MMCR differs conceptually from conventional linear extrusion measurements. Whereas medial meniscal extrusion quantifies the displacement of the meniscus beyond the tibial margin, MMCR is an area‐based measure that represents the proportion of the medial tibial cartilage covered by the meniscus. Therefore, the MMCR may reflect both the outward meniscal displacement and differences in meniscal morphology and tibial cartilage coverage. For this reason, direct quantitative comparison with previous studies using meniscal extrusion should be made with caution.

The association between tibial alignment and meniscal coverage appeared to differ according to the OA severity, as a positive MPTA–MMCR correlation was observed in KL Grades 3–4 knees but little evidence supported an association in KL Grades 0–2 knees. One possible explanation is that progressive meniscal degeneration, capsular or ligamentous changes, and cartilage loss may alter the relationship between bony alignment and meniscal position as OA advances. However, the MPTA‐by‐severity interaction was not statistically significant. Therefore, any apparent stage‐specific difference should be considered exploratory and requires confirmation in larger cohorts.

Traditional alignment evaluation relies primarily on weight‐bearing radiographic parameters, and full‐leg radiographs remain necessary for direct assessment of aHKA and %MA. The present findings do not suggest that MRI‐based measurements can replace radiographic alignment assessment or JLCA. Rather, when MRI is obtained for a clinical evaluation of intra‐articular pathology, the measurements of meniscal coverage and cartilage thickness may provide complementary structural information that is not captured by radiographic alignment parameters alone. Further studies are required to determine whether incorporating these MRI‐based measurements might improve clinical decision‐making beyond that achieved using conventional radiographic assessment.

In high tibial osteotomy (HTO) procedures, intra‐articular and joint‐line factors are increasingly recognized as important determinants of alignment correction. Micicoi et al. [14] reviewed the management of intra‐articular deformity with HTO, and failure to account for the joint‐line obliquity has been reported to result in tibial overcorrection [16]. Meniscal procedures have also been associated with postoperative alignment changes after HTO. Katagiri et al. [8] demonstrated that open‐wedge HTO combined with arthroscopic centralization of the MM resulted in a significant postoperative reduction in JLCA, although this finding does not establish an independent effect of meniscal repositioning on alignment correction. A recent systematic review reported improved objective outcomes after a combination of HTO and MM root repair compared with HTO alone [25], whereas a meta‐analysis found only limited additional clinical benefit from root repair [2]. Thus, the available evidence regarding the contribution of concomitant meniscal procedures remains mixed. Because the present study did not evaluate treatment, postoperative alignment or clinical outcomes, any potential relevance of the associations observed here to meniscal procedures during HTO should be regarded as hypothesis‐generating and will require prospective evaluation.

Several limitations of the present study warrant consideration. First, its cross‐sectional design precludes causal inference. The present data cannot establish (i) whether varus malalignment leads to loss of meniscal coverage and cartilage thinning, (ii) whether pre‐existing intra‐articular structural changes alter mechanical alignment, or (iii) whether both reflect the progression of OA. In addition, the interaction between MPTA and OA severity was not statistically significant; therefore, the apparent difference in the MPTA–MMCR association between severity strata should be regarded as exploratory.

A second limitation is the modest sample size, particularly that of the advanced OA subgroup. The KL Grade 3–4 subgroup included 33 knees, of which only six had KL Grade 4; therefore, the findings for advanced OA may not be fully generalizable to KL Grade 4 knees. Nevertheless, sensitivity analyses that excluded KL Grade 4 knees and used Spearman rank correlation yielded results generally consistent with the primary analyses. Five patients contributed bilateral knees, but restricting the analysis to one knee per patient produced similar results.

A third limitation involves the inherent limitation of the MRI‐based assessment of the MM. The MMCR is an area‐based measure of meniscal coverage rather than a direct measure of meniscal extrusion, and a lower MMCR may reflect several factors, including meniscal displacement, altered or reduced meniscal size, differences in tibial cartilage area or a combination of these. Moreover, because only the MM was evaluated, no individual assessment was made of meniscal tears, including root tears. Therefore, the potential influence of tears on meniscal position, coverage, and the observed associations could not be determined.

Limitations also arose related to imaging and measurement methodology. Lower limb alignment was assessed using weight‐bearing anteroposterior long‐leg radiographs, which primarily characterize coronal alignment and do not adequately assess rotational deformity or knee flexion contracture. Because flexion contracture may alter the projected coronal alignment and JLCA measurements, its potential influence cannot be excluded.

Finally, the study population was restricted to women in their 50s. This limits the generalizability of the findings to men, other age groups and broader populations with knee OA. Future studies that include more diverse populations will be required to determine whether the observed associations are reproducible across sex and age groups.

### Clinical relevance

The present findings suggest that radiographic alignment assessment alone may not fully characterize knees with OA, because meniscal coverage and cartilage thickness provide additional information regarding intra‐articular structural status. Accordingly, MRI‐based assessment of these structures may complement conventional radiographic evaluation in patients being considered for HTO. This type of combined assessment may contribute to a more comprehensive preoperative understanding of the factors associated with medial compartment alignment.

## CONCLUSION

Bony alignment and MRI‐based intra‐articular structural measurements were associated with weight‐bearing mechanical axis alignment in knee OA. When added to the aHKA, the MRI‐based meniscal coverage and cartilage thickness explained %MA with a model fit comparable to that obtained by adding JLCA. This finding suggests that these MRI‐based measurements may provide structural information that complements conventional radiographic evaluation.

## AUTHOR CONTRIBUTIONS

**Atsushi Sasabe**: Conceptualization; methodology; formal analysis; visualization; writing—original draft. **Nobutake Ozeki**: Investigation; project administration; supervision. **Hisako Katano**: Software; data curation; resources. **Yusuke Nakagawa**: Investigation. **Tomomasa Nakamura**: Investigation. **Hideyuki Koga**: Investigation. **Ichiro Sekiya**: Writing—review and editing; project administration; supervision. All authors have read and approved the final manuscript.

## FUNDING INFORMATION

The authors have no funding to report.

## CONFLICT OF INTEREST STATEMENT

The authors declare no conflicts of interest.

## ETHICS STATEMENT

This study was conducted in accordance with the Declaration of Helsinki. Ethical approval was granted by the Medical Research Ethics Committee of the Institute of Science Tokyo (approval number: M2023‐088). Informed consent was obtained from all individual participants included in the study.

## Supporting information

Supporting File 1.

## Data Availability

The data that support the findings of this study are available from the corresponding author upon reasonable request.
