# Supplementary material for: Magnetic resonance imaging‐based meniscal coverage and cartilage thickness are associated with mechanical axis alignment in knee osteoarthritis
Source: J Exp Orthop. 2026 Oct 3;13(4):e70927. doi: 10.1002/jeo2.70927 (PMC13633560; doi:10.1002/jeo2.70927)
Supplement: Supplementary file 1 [file JEO2-13-e70927-s001.docx]

**Supplementary Table 1.** Comparison of demographic characteristics, radiographic alignment indices, and MRI-based meniscal and cartilage measurements between the Kellgren–Lawrence (KL) grade 0–2 and 3–4 groups.

| **Variable** | **KL grade 0–2 (n=61)** | **KL grade 3–4 (n=33)** | **ANOVA *p*** | **2-group *p*** |
| --- | --- | --- | --- | --- |
| Height (cm) | 158.3 ± 5.9 | 157.8 ± 6.4 | 0.386 | 0.706 |
| Age (years) | 54.3 ± 2.7 | 55.3 ± 3.1 | 0.430 | 0.129 |
| BMI (kg/m²) | 24.7 ± 4.8 | 25.8 ± 4.4 | 0.630 | 0.266 |
| %MA (%) | 39.2 ± 15.0 | 30.3 ± 18.7 | 0.151 | **0.022*** |
| LDFA (°) | 87.5 ± 2.5 | 87.9 ± 1.9 | 0.864 | 0.433 |
| MPTA (°) | 86.3 ± 2.1 | 85.5 ± 1.8 | 0.474 | 0.069 |
| JLCA (°) | 0.6 ± 1.5 | 2.2 ± 2.5 | **0.002*** | **0.002*** |
| aHKA (°) | -1.3 ± 3.1 | -2.4 ± 2.7 | 0.528 | 0.071 |
| MMCR | 0.44 ± 0.12 | 0.38 ± 0.17 | **0.009*** | 0.080 |
| F (mm) | 1.97 ± 0.21 | 1.95 ± 0.21 | 0.157 | 0.711 |
| MF (mm) | 1.95 ± 0.27 | 1.71 ± 0.43 | **0.009*** | **0.004*** |
| LF (mm) | 1.87 ± 0.26 | 2.02 ± 0.37 | **0.028*** | **0.043*** |
| T (mm) | 2.15 ± 0.26 | 2.01 ± 0.36 | 0.099 | 0.053 |
| MT (mm) | 1.92 ± 0.30 | 1.65 ± 0.52 | **0.005*** | **0.009*** |
| LT (mm) | 2.44 ± 0.38 | 2.45 ± 0.59 | 0.889 | 0.976 |

Data are presented as mean ± standard deviation. The ANOVA *p*-value was derived from one-way analysis of variance across the five KL grades (0, 1, 2, 3, and 4). The 2-group *p*-value was derived from an unpaired Welch t-test comparing the KL grade 0–2 and 3–4 groups. Values shown for each group are the pooled mean ± standard deviation. Asterisks (*) indicate statistical significance (*p* < 0.05).

BMI, body mass index; %MA, percentage of mechanical axis; LDFA, lateral distal femoral angle; MPTA, medial proximal tibial angle; JLCA, joint line convergence angle; aHKA, arithmetic hip–knee–ankle angle; MMCR, medial meniscus coverage ratio; F, femoral cartilage thickness; MF, medial femoral cartilage thickness; LF, lateral femoral cartilage thickness; T, tibial cartilage thickness; MT, medial tibial cartilage thickness; LT, lateral tibial cartilage thickness.

**Supplementary Table 2.** Sensitivity analyses confirming the robustness of the main regression and correlation findings.

**(A) Regression models in the one-knee-per-patient dataset (89 knees) compared with the main analysis (94 knees).**

| **Regression model** | **Adjusted R² (89)** | **Adjusted R² (94)** |
| --- | --- | --- |
| Model A (aHKA) | 0.669 | 0.668 |
| Model B (aHKA + JLCA) | 0.839 | 0.840 |
| Model C (aHKA + cartilage difference + MMCR) | 0.843 | 0.841 |
| Combined (aHKA + JLCA + cartilage difference + MMCR) | 0.854 | 0.864 |

**(B) MPTA–MMCR correlation across subgroups, and robustness to exclusion of KL grade 4 knees and to Spearman rank correlation.**

| **MPTA–MMCR correlation** | **n** | ***r*** | ***p*** |
| --- | --- | --- | --- |
| Overall (KL 0–4), 89 knees | 89 | 0.211 | 0.047 |
| KL grade 0–2, 89 knees | 56 | 0.047 | 0.734 |
| KL grade 3–4, 89 knees | 33 | 0.404 | 0.020 |
| KL grade 3 only (KL 4 excluded), Pearson | 27 | 0.379 | 0.051 |
| KL grade 3 only (KL 4 excluded), Spearman | 27 | 0.378 | 0.052 |
| KL grade 3–4, Spearman | 33 | 0.382 | 0.028 |

Adjusted R² denotes the adjusted coefficient of determination. The one-knee-per-patient dataset retained the first-scanned knee for the five patients who contributed both knees. Pearson (or Spearman, where indicated) correlation coefficient.

MPTA, medial proximal tibial angle; MMCR, medial meniscus coverage ratio; aHKA, arithmetic hip–knee–ankle angle; JLCA, joint line convergence angle; KL, Kellgren–Lawrence.
